# General Surgery

**Published:** 1894-07-21

**Authors:** 


### GENERAL SURGERY.

**Fractures and Dislocations.**—As Gould remarks in a recent lecture[17] the treatment of fractures is a far more important matter than is frequently believed. Nature requires at every step the most intelligent and skilful aid at the hands of the surgeon. There is no fracture which does not demand the greatest care, and there are no cases which tax the resources of the surgeon more than do some cases of broken bones. The fragments should be replaced at once. It is an absolutely fatal mistake for the surgeon to content himself with the thought that he has put up the fracture temporarily and that he will put it up a little better when he takes it down. Reduction should be done at once and ought to be exact. Mr. Gould emphasises the fact that the real merit of plaster of Paris or other form of moulded splint when properly applied is due to its fitting equably to the whole limb and not to its rapidity of application. The time is not far off, as Mr. Lane[18] has brought evidence to prove in several cases of oblique fracture of the tibia and fibula in labourers, when the surgeon will feel it his duty in certain cases of simple fracture, if he cannot otherwise reduce the fragments accurately, to cut down upon the fracture and restore the parts to the position they were in before the accident. Although there are some advocates of the massage treatment of fractures without immobilisation who claim good results—equal, if not superior, to that obtained by the use of splints, extension, &c.—yet, as G. W. King says,[19] few would care about taking the risk of treating fractures without some retentive apparatus. Besides the advantages just mentioned, the treatment by plaster of Paris permits free access to the limb, and this allows us to combat successfully the degenerative changes in the muscles and to aid the rapid absorption of excessive effusion that prevents the normal process of healing.

For fracture of the jaw Gould[20] recommends Hammond's wire interdental splint. Failing this and other methods in a case in which the bone was fractured at both angles as well as a V piece from the chin, J. D. Thomas[21] used an inside and outside splint of soft steel with clamp welded to the inside one, which was placed behind and below the alveolar process. The outer one was fitted in front, sharp pins on the inner splint penetrate the alveolar process, and the screw of the clamp when tightened fastens the splints and fragments securely together. John Ashhurst, jun.,[22] discusses the diagnosis and treatment of fracture of the neck of the humerus. Dugas's test consists in placing the hand of the affected side upon the opposite shoulder, and at the same time bringing the elbow of the affected side in contact with the chest; if this can be done satisfactorily there is no dislocation. Three cases of dislocation in which resection of the upper end of the humerus was performed is the subject of a clinical lecture by Edmund Owen.[23] The first was an old unreduced subglenoid dislocation, the second a frequently recurring dislocation, and the third a subcoracoid dislocation with fracture through the anatomical neck. In each case the wound healed promptly. Owen alludes to the danger of forcible attempts at the reduction of old standing dislocations and the advantages of a clean incision with or without resection. Anything over eight weeks he would regard as an old standing dislocation. In the treatment of fracture of the humerus just above the condyles, Ashhurst[24] employs in the earlier stage the right angled anterior splint, which has the advantage of overcoming any tendency to recurrence of the deformity; in the later stages he uses an internal angular splint, often supplemented with a pasteboard cap to the outer side of the arm. C. A. Powers[25] raises the question whether the treatment of fractures at the elbow by complete extension is ever proper. It seemed to him that the method is not to be recommended to the general practitioner; within the last two years two cases so treated had come under his observation with ankylosis, unreduced dislocation and almost entire disability, so that arthrotomy had to be performed in both. A. T. Hudson[26] states that his brother worked out, twenty years ago, the diagnosis of fracture of the neck of the femur by means of one symptom. A person in good health and over forty receives a fall. He is disabled and cannot get up without assistance. This means nothing less than a broken bone, and invariably a fractured neck of femur. In five cases of fractured neck of femur in old age Hudson employed extension by weight and pulley, and supported the hip and leg by sandbags; a pillow under the knee was sometimes needed to put the limb on an inclined plane. Elevation of the foot of the bed was sufficient for counter-extension. W. W. Browning[27] draws attention to the influence of the ilio-psoas muscle upon the rotation of the thigh. His conclusions agree with those given by Davies-Colley in Morris's "Anatomy," viz., that on account of the angle which the neck of the femur forms with its shaft, the point of attachment of this muscle is really external to the axis of rotation, and that therefore any power of rotation exercised by the muscle will be rather internal than external. Browning formulates the rule: All muscles attached to the femur which have their fixed point internal to the axis of rotation rotate the bone internally, provided the line of traction passes anterior to such axis. If the line of traction passes posterior to such axis, the rotation will be external. Schmid[28] treats fracture of the leg in the following way. The bones are set by extension, counter-extension, and manipulation, and the limb placed upon a splint, elevated, and cold applied. After the swelling has gone down, in three to six or eight days, a plaster of Paris bandage is applied during extension, and the patient allowed to stand and walk the following day. Five weeks later the bandage is removed and massage commenced. All his patients are said to have done well, without deformity or complication. The plaster of Paris may be applied immediately if the patient is seen directly after the accident. For ordinary fracture of the leg or ankle, W. Meacher[29] has used for the past fifteen years a light but yet strong splint of perforated sheet zinc, with a foot piece which holds the foot perfectly, and protects the toes from the weight of the bed-clothes. It also encircles and covers the limb sufficiently to hold it perfectly secure when bandaged. To examine the fracture at any time, the side of the splint may be easily bent out after removing the bandage, and an opening may be made in any part for compound fracture. P. Caviglia[30] records an interesting case of fracture of the tibia in the fœtus from a blow on the abdomen at the fifth month of pregnancy. The leg was bent at an angle and smaller than the other, the foot being in the equino-varus position. Over the site of fracture was a cutaneous cicatrix, and no trace of a fibula. Linear osteotomy and tenotomy were performed twelve days after birth, with improvement in the state of the limb. The third lecture at the Royal College of Surgeons on traumatic separation of the epiphyses, by Jonathan Hutchinson, jun., gives[31] a short review of those of the lower extremity; and A. H. Tubby has given[32] a similar collection with notes. The *Lancet* publishes[33] a summary of the accidents on the football field which have been reported during the last quarter of 1893 and during January, February, and March, 1894. In the former period there were 2 concussions of the spine, 1 concussion of brain, 1 fracture of thigh, 16 fractures of leg, 5 fractures of arm, and 5 deaths; in the latter period, 1 concussion of brain, 15 fractures of leg, 6 fractures of collar bone, 1 fracture of shoulder blade, 1 fracture of arm, 5 dislocated shoulders, 1 dislocated knee, 6 undescribed hospital cases with 3 deaths, 5 deaths. These lists, though imperfect, indicate that football is dangerous; of late the dangers have in some directions increased and in others diminished.

[17] Clinical Journal Jan. 17, 1894, p. 179. [18] Brit. Med. Journal, April 21, 1894. [19] Medical Reprints, Jan. 15, 1894, p. 184. [20] Clinical Journal, Jan. 17, 1894, p. 185. [21] Internat. Med. Mag., Jan., 1894, p. 1,108. [22] Inter. Med. Mag., Dec., 1893, p. 1,028. [23] Clinical Journal, Feb. 28, 1894, p. 273. [24] Inter. Clinics, vol. iii., p. 195. [25] Annals of Surgery, Feb., 1894, p. 236. [26] N. Y. Med. Record, Feb. 24, 1894, p. 234. [27] Annals of Surgery, Feb., 1894, p. 223. [28] Amer. J. Med. Sci., Feb., 1894, p. 200, and Centralbl. für Chirurg., No. 32. [29] N. Y. Med. Record, Feb. 17, 1894, p. 140. [30] Ed. Med. Journal, Feb., 1894, and Ann. di Ostetr. e Ginecol. [31] Brit. Med. J.. March 31, 1894, p. 670. [32] Annals of Surgery, March, 1894, p. 289. [33] Lancet, March 24, 1894, p. 764.

